# Research on the Hydration Mechanism of Active Roof-Contact Backfill Materials: Effect of Expansive Agent Types and Dosages

**DOI:** 10.3390/ma19040662

**Published:** 2026-02-09

**Authors:** Zepeng Yan, Xun Chen, Guoqiang Wang, Shenghua Yin, Lijie Guo, Caixing Shi, Shishan Ruan, Jialu Zeng

**Affiliations:** 1 BGRIMM Technology Group, Beijing 100160, China; 2 School of Resources and Environmental Engineering, Shandong University of Technology, Zibo 255049, China; 3 Xinjiang Kalatongke Mining Co., Ltd., Fuyun 836107, China; 4 School of Civil and Resources Engineering, University of Science and Technology Beijing, Beijing 100083, China; 5 National Centre for International Research on Green Metal Mining, Beijing 100160, China

**Keywords:** active roof-contact, mine filling, roof control, expansive agent, heat release, isothermal calorimeter, hydration mechanism

## Abstract

Failure to fully backfill the goaf may result in increased exposure of roof strata, significantly raising the risk of roof collapses in mining zones and potentially causing surface subsidence, thereby endangering the safety of mining personnel. To address this issue, expansive agents are utilized to produce active roof-contact backfill (ARCB) materials, which promote localized self-compaction of backfill materials in unroof-contact areas through hydration reactions. In this study, an isothermal calorimeter was employed to measure the ARCB hydration heat release rate curves of three types of expansive agents, CaO-based, MgO-based, and ettringite-based, at dosages ranging from 6% to 12%. Hydration kinetic parameters were calculated based on the Krstulovic–Dabic model. The influence of expansive agent type and dosage on these parameters was analyzed, and the hydration mechanism of ARCB materials was investigated. The results indicate that the hydration process of grouting materials using all three expansive agents follows five distinct stages: rapid reaction, induction, acceleration, deceleration, and decay. However, increasing the dosage of the CaO-based expansive agent will enhance heat release and prolong the duration of the acceleration stage. When the dosage is 12%, the total heat release reaches 327.4 J·g^−1^. At the same dose, the sample doped with MgO-based expansive agent was only 254.3 J·g^−1^, which was 22.3% lower than that of CaO-based, and the occurrence time of the second heat release peak was earlier. In contrast, the ettringite-based expansive agent shows a decreasing trend in heat release with increasing dosage. Furthermore, the use of CaO-based and MgO-based expansive agents allows the hydration process to bypass the phase boundary reaction (I) stage and directly enter the diffusion (D) stage. Ettringite-based expansive agents still undergo three stages, but exhibit a shortened nucleation and growth (NG) stage and an extended induction stage. Additionally, different expansive agents have varying effects on the crystal growth index (n), reaction rate constant, and degree of hydration.

## 1. Introduction

Mineral resources serve as the material foundation of modern industrial society, continuously ensuring the supply of raw materials required for socio-economic development [1,2,3,4,5,6]. Long-term intensive mining has led to the gradual depletion of high-quality shallow-level resources, making the development of deep-level mineral resources an inevitable choice for the sustainable development of the global mining industry and a critical strategic measure to ensure national resource security [7,8,9]. Industry data indicates that within the next fifteen years, more than half of China’s iron ore, coal, and certain non-ferrous metal mines will have mining depths exceeding 1 km [10,11]. The complex deep-earth environment will exert a significant influence on the roof of the goaf. High geothermal temperatures will significantly weaken rock strength, accelerate its deformation process, and make rock layers more prone to deformation and instability [12]. Additionally, the strong coupling of high geothermal temperatures and high geological stresses induces a 3- to 8-fold increase in the frequency of dynamic disasters such as large-scale deformation of surrounding rock and rock bursts compared to shallower depths, posing a severe threat to the safe extraction of deep-seated resources [13,14,15,16,17].

Filling mining methods, which combine rock pressure control, rock displacement suppression, and solid waste resource utilization functions, have become the core mining method for deep high-risk ore bodies [18,19,20]. Engineering practice has shown that, due to objective conditions such as bleeding and settlement of the fill slurry, material segregation, and irregularities in the mine roof, the fill body often fails to effectively reach the roof [21]. Backfill roof contact quality is a core technical indicator for evaluating backfill effectiveness in mines, directly impacting the stability of deep mining operations [22]. Roof contact rate, as a key quantitative parameter for backfill roof contact quality, is typically defined as the ratio of the effective contact area between the backfill body and the roof, or alternatively represented by the ratio of backfill volume to the volume of the goaf [23,24]. Its value is directly related to the geometric dimensions of the exposed roof area. When it falls below the critical threshold, it triggers rock mass system instability, leading to roof collapse, redistribution of surrounding rock stress, and chain failure of the backfill structure, posing a significant safety threat to underground workers [25,26]. Therefore, improving the contact quality between the filling body and the roof is a key technical bottleneck for ensuring the safe and efficient mining of deep mineral resources.

From an engineering practice perspective, beyond the inherent issues mentioned above, the core engineering challenge in achieving effective contact between deep backfill and the roof lies in the coupled effects of high geothermal heat and high ground stress in deep underground environments. These factors significantly increase the difficulty of controlling roof stability, substantially reducing the applicability of conventional backfill techniques. To address this challenge, academia and the engineering community have developed multiple filling and roof support technologies centered on achieving effective contact between the fill and the roof. These specifically include multiple-stage filling techniques, active roof collapse control technologies, grout pressure regulation methods, and gravity grouting roof support systems [27,28]. However, these techniques all fall under the category of “forced roof contact,” with the core logic being to compel the backfill to adhere to the roof through external force or process optimization. In practical applications, they commonly exhibit significant shortcomings. First, the construction process is complex, requiring substantial additional investment in equipment and manpower, thereby increasing mining costs. Second, the roof contact effect is unstable, being highly influenced by factors such as roof morphology and grout fluidity, making it difficult to adapt to complex and variable geological conditions at depth. Third, they lack adaptability, exhibiting insufficient compatibility with ore bodies of different mineral types and mining depths, and failing to fundamentally resolve the challenge of roof-contact in deep-level backfilling [10,29].

Expansion filling is a new method in the field of mine filling in recent years. Due to its strong water retention capacity, fast setting speed, and active roof contact, it has gradually been applied in production practice. Shi [30] added metal powder to the grout as a self-expanding material. The metal powder reacts chemically with water in an alkaline environment to generate gas. These bubbles can be uniformly suspended in the grout, forcing the grout to expand in volume. Yu [31] used hydrogen peroxide as a foaming agent. The silica cement in the grout accelerates the chemical reaction rate of H_2_O_2_, producing a large amount of gas in a short time, thereby increasing the volume of the grout. However, the mass and physical–chemical properties of the grout itself remain unchanged. Wang [32] proposed replacing part of the traditional cementitious materials with static fracturing agents. The main component of static fracturing agents is CaO, which reacts with water to form Ca(OH)_2_, expanding in volume by 4–5 times. However, from the perspective of engineering application effectiveness, existing expansion technologies still exhibit significant shortcomings: gas-phase expansion agents markedly reduce the strength of the filling material, and their excessively rapid reaction rates hinder pipeline transportation. Meanwhile, solid-phase expansion agents suffer from insufficient stability in their expansion effects and limited adaptability, and have yet to establish mature engineering applications.

Starting from the essence of materials science, the core of expansive filling technology lies in the synergistic effect of expansive agents with cementitious materials, tailings, and other components, which achieves controllable volume expansion through hydration reactions. This ensures that the filling body has sufficient mechanical strength to withstand the pressure of the surrounding rock. Therefore, the hydration characteristics of the expansive agent are the core scientific element that determines the filling effect. For example, Cao [33] studied the effects of MgO activity and dosage on the expansive and mechanical properties of mortar, finding that depending on curing temperature and dosage, expansive agents can have either a positive or negative impact on mortar expansion. Xiao [34] found that the addition of calcium-based expansive agents increases the hydration rate and heat of hydration during the accelerated phase of composite cementitious systems and delays the volcanic ash reaction process of slag powder. Existing research has primarily focused on pure mortar systems, failing to consider practical filling components such as tailings or the impact of high-geothermal environments. Furthermore, no quantitative relationship has been established between the hydration kinetics parameters of expansive agents and the macroscopic properties of the fill material. This lack of a theoretical foundation hinders the optimization of engineering technologies.

In summary, the disconnect between engineering technical deficiencies and gaps in materials science knowledge represents the key bottleneck constraining solutions to deep backfill roof contact challenges. This study aims to reveal the hydration kinetics of CaO-based, MgO-based, and calcined gypsum-based expansive agents within the cementitious material-tailings system. It establishes correlations between expansive agent type/dosage and hydration characteristics as well as macroscopic performance, providing theoretical support for deep “active capping” backfill technology. The originality of this research lies in its application to actual backfill systems, addressing the fundamental hydration kinetics to achieve precise integration between materials science understanding and engineering optimization, demonstrating significant research value.

## 2. Materials and Methods

### 2.1. Materials

#### 2.1.1. Tailing

**Figure 1:**
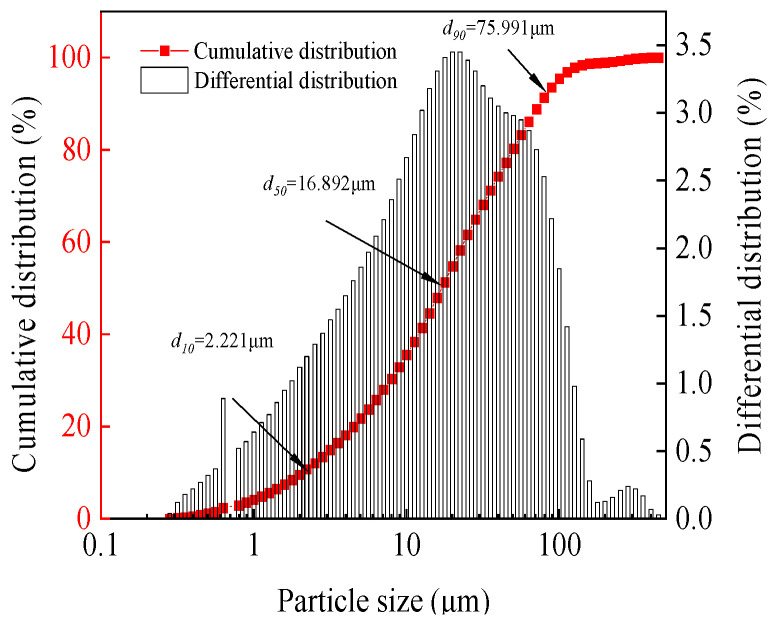
The particle size distribution curve of the tailings.

The tailings used in this study were sourced from a nickel mine in Gansu Province. Among them, after the tailings were dried at high temperature (105 ± 3 °C), their specific gravity was measured to be 2.658. Figure 1 presents the tailings particle size distribution data obtained by the laser particle size analyzer (LMS-30, Seishin Enterprise Co., Ltd., Tokyo-to, Japan). The particle size range of the tailings is mainly distributed between 10.0 and 80.0 μm, among which the characteristic particle sizes d_10_, d_50_, and d_90_ are 2.221 μm, 16.892 μm, and 75.991 μm, respectively. At the same time, X-ray fluorescence (XRF) determination was also conducted on the tailings, as shown in Table 1. It can be seen that the main chemical components of tailings are SiO_2_ and MgO, and the content of elements (S and P) that affect strength development is relatively low.

#### 2.1.2. Expanding Agent (EA)

**Figure 2:**
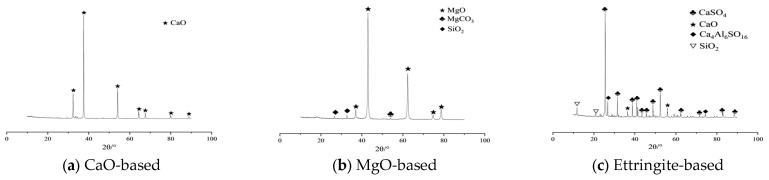
XRD patterns of EA.

Based on the literature review, this study selected three chemical reaction-type solid-phase EA suitable for mine filling, namely MgO-based, CaO-based, and ettringite-based, all of which were available on the market. Among them, the activity index of the MgO-based EA measured by the citric acid method was 110 s. Table 2 lists the chemical compositions of the three EA materials, and Figure 2 shows their XRD spectra. Table 2 and Figure 2 show that the main component of the CaO-based EA is unburned CaO particles, which will produce a large amount of Ca(OH)_2_ crystals when in contact with water. The hydration of MgO-based EA will form (Mg(OH)_2_). The ettringite-based EA is mainly composed of calcium sulfoaluminate (C_4_A_3_S) and CaO, and it will rapidly form ettringite during the hydration process.

#### 2.1.3. Binder and Water

Ordinary Portland cement (P.O 32.5), which is commonly used in mines, was chosen as the binder for this experiment. In addition, local tap water was used to prepare the ARCB samples.

#### 2.1.4. Sample Preparation

To study the mechanism of action of EA type and dosage on filling materials, we conducted a comprehensive experiment based on the parameters in Table 3. Among them, the concentration of the filling slurry is 78%, and the dosage of the expansive agent is based on the proportion of cement usage.

### 2.2. Hydration Behavior Test

**Figure 3:**
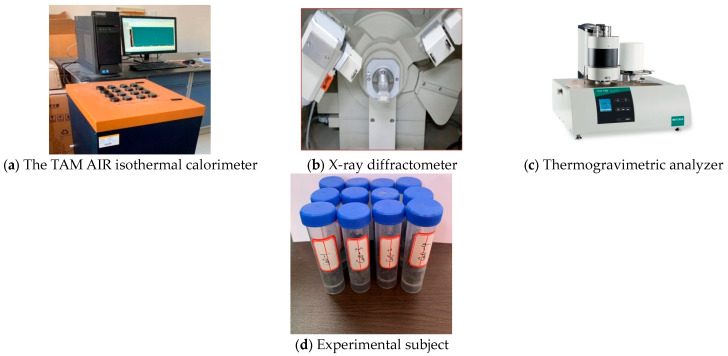
The test equipment used in the study.

**Figure 4:**
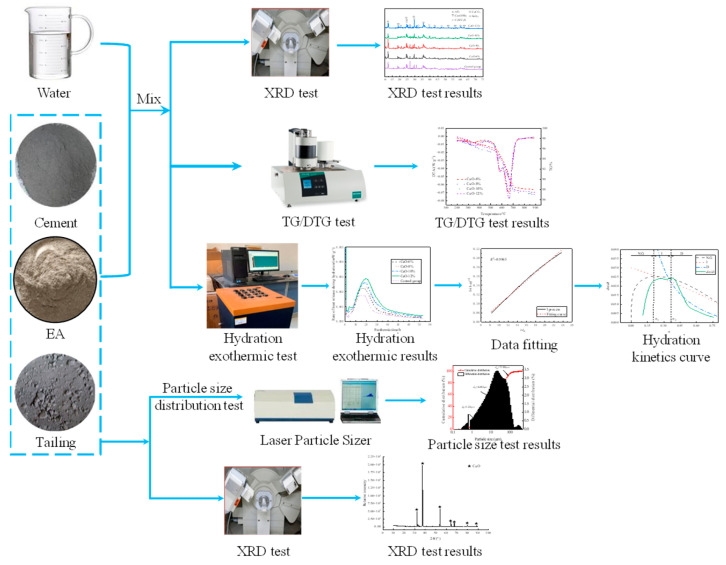
The flowchart-style outline of the research program.

The early hydration test used a TAM AIR cement isothermal calorimeter manufactured by TA Instruments in the United States (New Castle, DE, USA), as shown in Figure 3a. It can test eight sets of samples simultaneously, with a test temperature range of 5 °C to 90 °C. Mid-to-late-stage hydration behavior (28 d) was tested using an X-ray diffractometer (STOE/2, STOE & Cie GmbH, Hessen, Germany) and a thermogravimetric analyzer (TGA55, TA Instruments, DE, USA), with the thermogravimetric testing range spanning 30 °C to 900 °C. The test content includes the early hydration and mid-to-late hydration behavior of pre-stressed materials. The specific experimental process is shown in Figure 4.

## 3. Early Hydration Mechanism

### 3.1. Principles and Models of Hydration Kinetics

#### 3.1.1. Principle of Hydration Kinetics

Hydration kinetics takes the rate of chemical reactions as its object, mainly studying and explaining the chemical reaction processes and rate changes under the combined action of internal and external factors [35,36]. The Arrhenius equation can be used to represent the relationship between the rate constant and temperature, as shown in Equation (1).

(1) $$K=A\cdot \exp\left(-\frac{E_{a}}{RT}\right)$$

The isothermal dynamic equation of the filling material is shown in Equation (2).

(2) $$d\alpha /\mathrm{dt}=K(t)\cdot f(\alpha )=A\cdot f(\alpha )\cdot \exp\left(-E_{a}/RT\right)$$

This kinetic model can be understood as characterizing the transformation rate of the reaction system by the product of the temperature-dependent rate constant *K*(T) and the *α* correlation function *f*(*α*).

#### 3.1.2. Hydration Kinetics Model

The Krstulovic–Dabic model is used to explain the hydration process of filling materials, which can be divided into the following stages [37]: crystallization, nucleation and crystal growth (NG), phase boundary reaction (I), and diffusion (D), and the final chemical reaction process of the filling material depends on the slowest stage. The equations and their differential forms of the aforementioned three reactions are as follows:

(1) Crystallization and nucleation:

(3) $${\left[-\ln(1-\alpha )\right]}^{1/n}=K_{1}\left(t-t_{0}\right)=K_{1}^{\prime }\left(t-t_{0}\right)$$

(2) Phase boundary reaction:

(4) $${\left[1-{\left(1-\alpha \right)}^{1/3}\right]}^{1}=K_{2}r^{-1}\left(t-t_{0}\right)=K_{2}^{\prime }\left(t-t_{0}\right)$$

(3) Diffusion:

(5) $${\left[1-{\left(1-\alpha \right)}^{1/3}\right]}^{2}=K_{3}r^{-2}\left(t-t_{0}\right)=K_{3}^{\prime }\left(t-t_{0}\right)$$

NG-process differential expression:

(6) $$d\alpha /dt=F_{1}(\alpha )=K_{1}^{\prime }n(1-\alpha ){\left[-\ln(1-\alpha )\right]}^{n-1/n}$$

I-process differential expression:

(7) $$d\alpha /dt=F_{2}(\alpha )=K_{2}^{\prime }\cdot 3{\left(1-\alpha \right)}^{2/3}$$

D-process differential expression:

(8) $$d\alpha /dt=F_{3}(\alpha )=K_{3}\cdot 3{\left(1-\alpha \right)}^{2/3}/\left[2-2{\left(1-\alpha \right)}^{1/3}\right]$$

where *α* represents the degree of hydration, *n* is the geometric crystal growth index, *t* represents the hydration time, $t_{0}$ it is the end time of the induction period, *r* is the radius of the reactant particles, $K_{i}$ is the reaction rate constant, $K_{i}^{\prime }$ is the apparent reaction rate constant, and $F_{i}(\alpha )$ represents the reaction mechanism function.

The experimental data can be converted into the required parameters *α* and *dα*/*dt* by using the formula proposed by Knudson [38,39].

(9) $$\frac{1}{Q(t)}=\frac{1}{Q_{\max}}+\frac{t_{50}}{Q_{\max}\left(t-t_{0}\right)}$$

where *Q*(*t*) represents the heat release at a certain moment *t* during the acceleration period, *Q_max_* represents the final total heat release, *t*_50_ is the time required to reach 50% of the total heat release [36], and (*t* − *t*_0_) represents the duration of hydration calculated from the start of the acceleration stage.

(10) $$\alpha (t)=\frac{Q(t)}{Q_{\max}}$$

(11) $$\frac{d\alpha }{dt}=\frac{dQ}{dt}\cdot \frac{1}{Q_{\max}}$$

### 3.2. Hydration Exothermic Property

**Figure 5:**
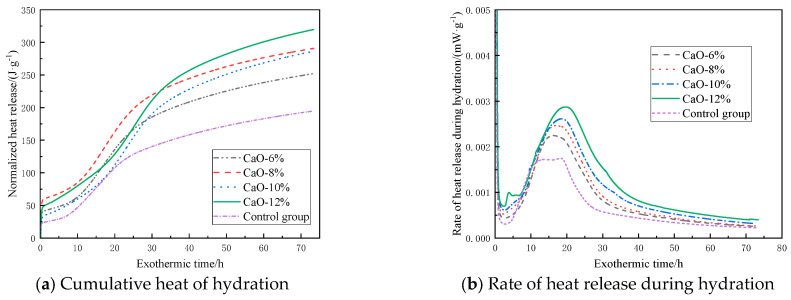
Heat release characteristics of filling materials doped with CaO-based expansion agent.

Figure 5 shows the hydration exothermic characteristics of the filling material containing CaO-based expansive agents. As shown in Figure 5a, the hydration reaction of the filling material after adding the CaO-based expansive agent is similar to that of the control group. Under different CaO-based expansive agent content conditions, the cumulative hydration heat curves all exhibit a trend of rapid increase followed by a slow rise, and as the addition of CaO-based expansive agent increases, the cumulative hydration heat also increases accordingly. Based on the data in Figure 5b, the hydration process can be divided into five stages:

(1) Rapid reaction stage. The filling material reacts with water, forming the first exothermic peak on the curve within 10 min.
(2) Induction stage. During this stage, the hydration reaction rate of the filling material is low, primarily depending on the hydration process on the surface of the material particles.
(3) Acceleration stage. During this stage, the hydration rate of the filling material rapidly increases as free water enters the interior of the particles for hydration.
(4) Deceleration phase. During this phase, the hydration reaction rate of the filling material gradually decreases, forming the second exothermic peak on the curve together with phase 3.
(5) Decay phase. The hydration reaction rate approaches 0.

As shown in Figure 5b, as the hydration process continues, all sample groups gradually reach the peak exothermic rate between 15 h and 21 h, after which they enter the deceleration phase. Compared with the control group, the time for the exothermic hydration rate to reach its peak is delayed when CaO-based expansive agents are added. However, as the addition amount increases, the duration of the acceleration phase also extends. For example, when the addition amount is 6%, the acceleration phase lasts 7.6 h, while when the addition amount increases to 12%, the acceleration phase extends to 13.4 h. As the dosage of the expansive agent increases, the CaO content in the system increases, continuously reacting with free water in the system to form Ca(OH)_2_ and generate a large amount of heat, thereby accelerating the hydration reaction. This results in a longer acceleration phase and higher heat release as the dosage increases.

**Figure 6:**
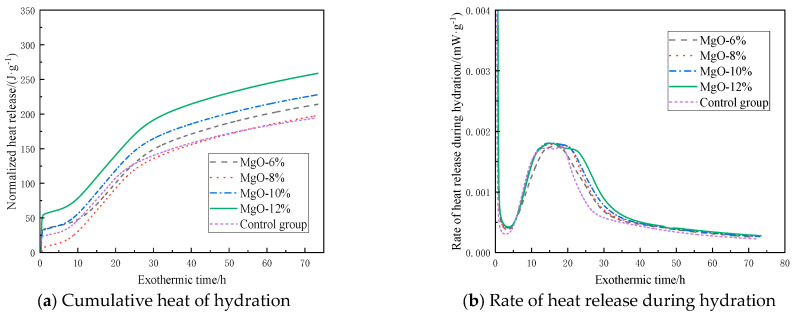
Heat release characteristics of filling materials doped with MgO-based expansion agent.

When switching to MgO-based expansive agents, as shown in Figure 6a, the hydration exothermic process of the filling material after adding MgO expansive agents is similar to that of CaO-based agents, but the final exothermic value is slightly lower. Taking a 12% addition rate as an example, the heat release of samples with CaO-based expansive agent is 327.4 J·g^−1^, while that of samples with MgO-based expansive agent is only 254.3 J·g^−1^. Under the same addition rate conditions, the heat release is 22.3% lower than that of the CaO-based expansive agent. Figure 6b shows that the first exothermic peak appears within 10 min, and as the hydration process continues, the samples gradually reach the peak exothermic rate between 12 and 17 h, after which the hydration rate begins to decrease. Compared to CaO-based expansive agents, the time for the second exothermic peak to appear is significantly reduced. After replacing a certain amount of cement with MgO-based expansive agents, the hydration reaction rate is reduced, leading to decreased heat generation in the system and thereby affecting the overall heat release characteristics. Additionally, compared to the control group, the samples containing MgO-based expansive agents had a slightly longer induction period, as the lower Ca^2+^ concentration in the pore solution required more time to reach saturation. The slightly higher heat release rate during the decay period compared to the control group was primarily due to the later hydration of MgO.

**Figure 7:**
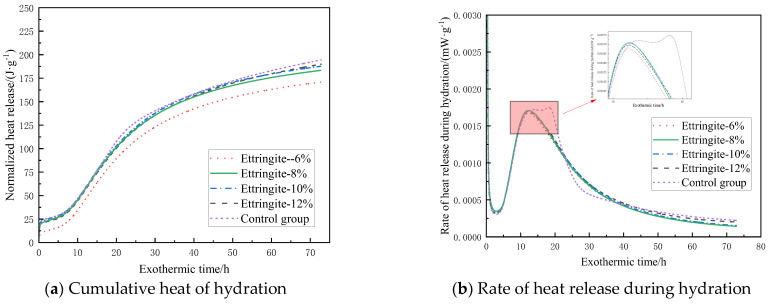
Heat release characteristics of filling materials doped with ettringite-based expansion agent.

Figure 7 shows the cumulative hydration heat and hydration heat release rate curves over time under different ettringite-based expansive agent dosage conditions. Figure 7a shows that increasing the expansive agent content leads to a gradual decrease in the total hydration heat release, with reductions of 0.86%, 2.3%, 5.67%, and 12.1% compared to the control group. This is primarily due to the consumption of a large amount of free water by calcium sulfoaluminate during hydration. As the dosage increases, the consumption of free water also increases, which inhibits the hydration of clinker in the system, thereby reducing heat release. Figure 7b shows that the peak value of the second heat release peak on the curve decreases with increasing dosage, with reductions of 4.21%, 3.17%, 2.66%, and 2.28% compared to the control group. Additionally, it was found that samples containing ettringite-based expansive agents reached the highest hydration rate and ended the induction period slightly earlier than the control group, and the exothermic rate during the decay period was lower than that of the control group, indicating that the addition of such expansive agents inhibits the reaction rate of the hydration system.

### 3.3. Analysis of Hydration Kinetics Process

**Figure 8:**
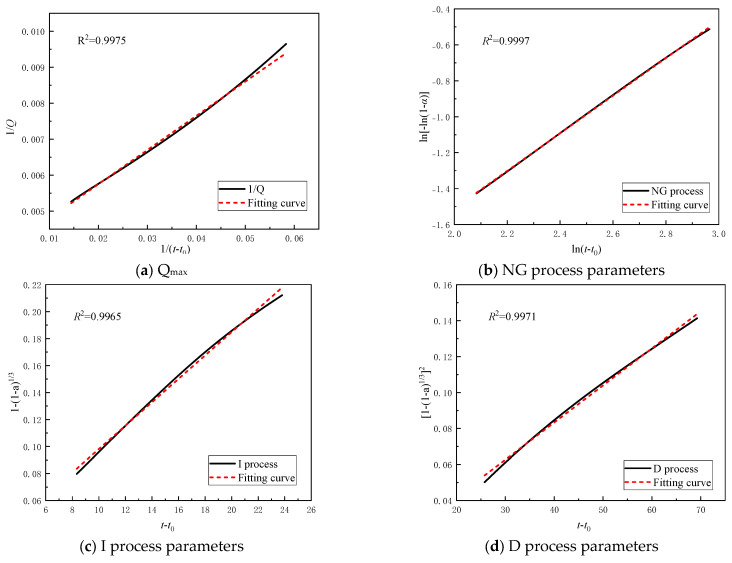
Parameter calculation.

According to Equation (9), $Q_{\max}$ and $t_{50}$ can be obtained by solving the linear fit of the hydration heat data, as shown in Figure 8a. After obtaining $Q_{\max}$, *α* and *dα*/*dt* can be obtained according to Equations (10) and (11). Given *α*, the kinetic parameters for different reaction processes can be obtained by fitting according to Equations (3)–(5), as shown in Figure 8b–d. Based on the fitted parameters, the relationships between $F_{1}(\alpha )$, $F_{2}(\alpha )$, and $F_{3}(\alpha )$ and the hydration degree *α* can be determined.

**Figure 9:**
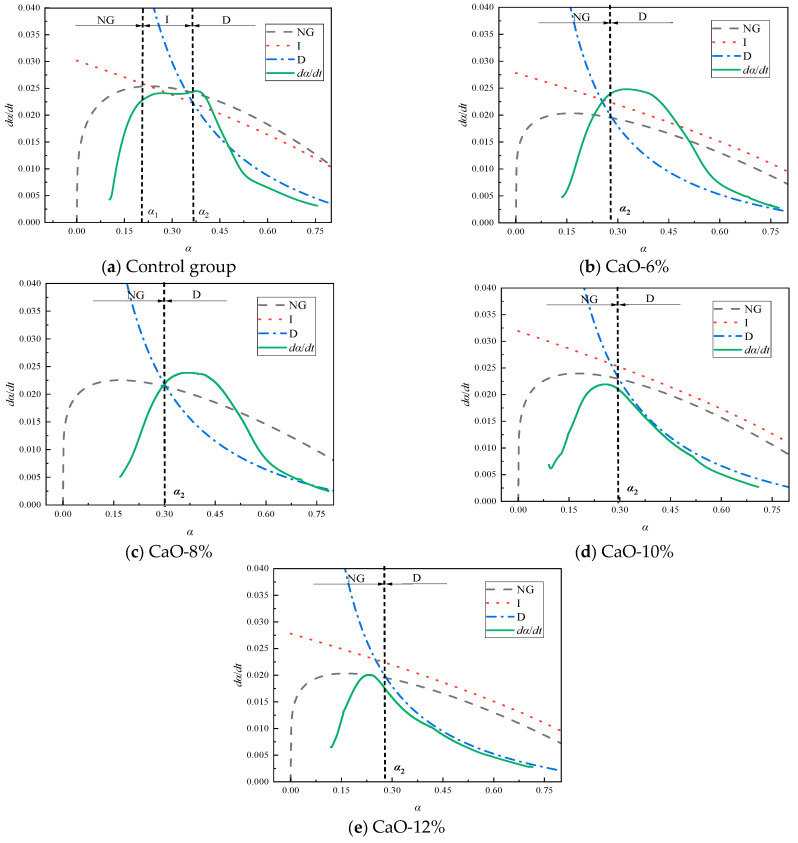
Hydration reaction rate curve of filling materials doped with CaO-based expansion agent.

Based on the previous exothermic cumulative curve, the exothermic heat released before the induction period is relatively low, accounting for approximately only 5% of the total exothermic heat. This process can be neglected in the overall reaction process of the filling material. Therefore, this study only analyzes the process after the induction period. Figure 9 shows the simulated curves and corresponding hydration rate curves calculated using Equations (6)–(8) under different CaO-based expansive agent content conditions. It can be observed that the hydration process of the filling material is simulated in segments by the Krstulovic–Dabic model. Compared with the control group, samples containing CaO-based expansive agents bypass the phase boundary reaction (I) and directly transition from the NG process to the D process. Figure 9 indicates that the hydration process of the filling material is not a single-step process but is controlled by multiple reaction mechanisms, and the decisive process is often the stage with the lowest rate. It was also found that the hydration reaction of the control group without expansive agents sequentially passed through the NG, I, and D stages. However, with the addition of CaO-based expansive agents, the filling samples only passed through the NG and D stages. This is because the addition of CaO promotes the early hydration process, leading to the rapid development of the NG process, which causes a sharp increase in ion migration resistance and thus bypasses the control of the I process.

Table 4 presents the hydration kinetic parameters of CaO-based expansive agent samples, where *n* denotes the crystal geometric growth index, $K^{\prime }$ represents the hydration rate values at different stages of the process, and α indicates the degree of hydration. From the data in Table 4, it can be seen that $K_{1}^{\prime }$ is approximately 2.6 times that of $K_{2}^{\prime }$ and approximately 14.3 times that of $K_{3}^{\prime }$. This indicates that the reaction rates of processes I and D are significantly lower than those of the NG process, attributed to the self-catalytic reactions occurring in the NG process, resulting in rapid formation and high yield of hydration products.

The dosage of CaO-based expansive agent increased from 6% to 12%, resulting in increases in the reaction order *n* of 0.95%, 1.72%, and 2.74%, respectively. The reaction rate constant $K_{1}^{\prime }$ increased by 13.4%, 26.05%, and 34.61%, respectively. Reaction rate constant $K_{2}^{\prime }$ increased by 1.98%, 19.14%, and 17.05%, respectively, and reaction rate constant $K_{3}^{\prime }$ increased by 1.19%, 23.21%, and 27.98%, respectively. The gradual increase in *n* values indicates that the CaO expansive agent promotes crystal growth. The gradual increase in the $K_{2}^{\prime }$ value indicates that increasing the dosage of the CaO expansive agent enhances the alkalinity of the pore solution, thereby accelerating the crystal growth rate. The increase in the $K_{3}^{\prime }$ value indicates that increasing the dosage of the CaO expansive agent increases the concentration of Ca^2+^ ions in the solution, reducing the time required to reach supersaturation and increasing the interfacial area. An increase in the value of 6 indicates that increasing the dosage of the CaO expansive agent enhances the diffusion ability of Ca^2+^ ions. Additionally, it was found that the rate constants under different dosage conditions exhibit a trend of $K_{1}^{\prime }$ > $K_{2}^{\prime }$ > $K_{3}^{\prime }$, attributed to the NG process being in the acceleration phase of the hydration reaction, the I process being in the acceleration and deceleration phases, and the D process being in the deceleration and stable phases.

The data in Table 4 also indicate that increasing the addition of CaO-based expansive agents leads to a gradual decrease in hydration degree. This is because CaO-based expansive agents enhance the hydration reaction in Stage D while reducing the reaction in Stage I, indicating that the addition of CaO-based expansive agents causes the reaction process to shift from NG to D under conditions of lower hydration degree. The addition of CaO-based expansive agents reduces the overall hydration degree of the system. Within the scope of this study, the hydration degree of the system decreases gradually as the dosage of CaO-based expansive agents increases.

**Figure 10:**
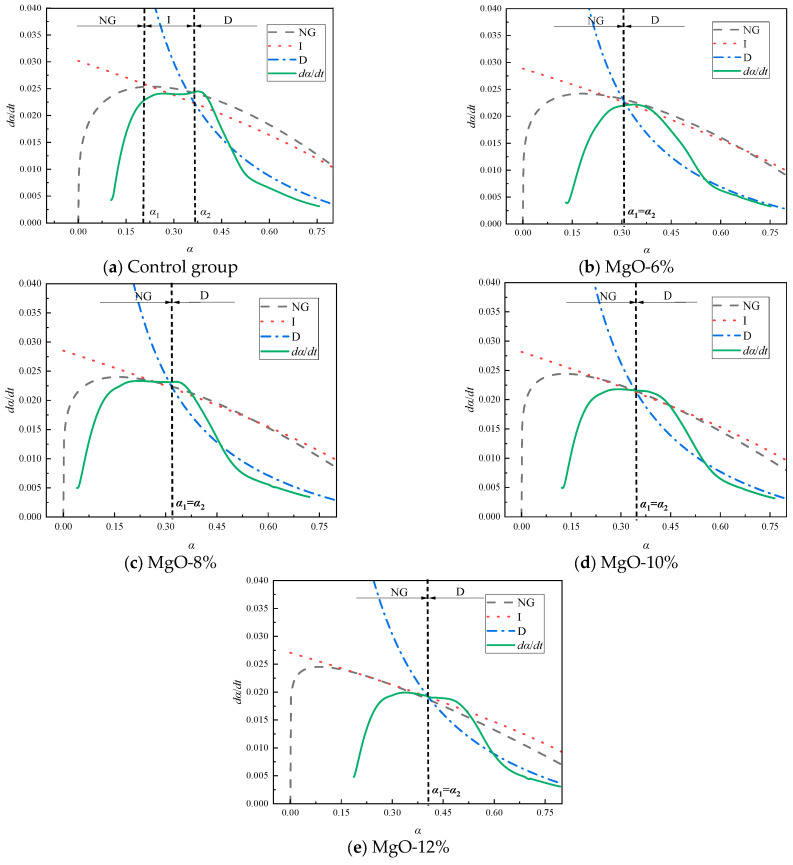
Hydration reaction rate curve of filling materials doped with MgO-based expansion agent.

Figure 10 shows the simulated curves calculated using the formula and the corresponding hydration rate curves under different MgO-based expansive agent content conditions. It can be seen that as MgO-based expansive agent is added, the fitted curves of the NG, I, and D stages intersect at a single point, indicating that the hydration system directly transitions from the I stage to the D stage without passing through the I stage. When MgO expansive agent is added, the CaO content in the system decreases, leading to rapid cement hydration and the deposition of hydration products on the particle surface. At this point, due to the limited Ca(OH)_2_ content, further hydration reactions are hindered, causing the reaction to directly transition from the NG stage to the D stage.

Table 5 lists the hydration kinetic parameters of the filling material containing MgO-based expansive agents. As shown in the data from Table 5, the value of $K_{1}^{\prime }$ is approximately 3.4 times that of $K_{2}^{\prime }$ and approximately 14.6 times that of $K_{3}^{\prime }$. As the addition of MgO-based expansive agents increases, the values of *n*, $K_{1}^{\prime }$, and $K_{2}^{\prime }$ gradually decrease, while the values of $K_{3}^{\prime }$, $\alpha_{1}$, and $\alpha_{2}$ gradually increase. Specifically, the reaction order *n* decreased by 3.07%, 6.97%, and 12.12%, respectively. The reaction rate constants $K_{1}^{\prime }$ decreased by 1.93%, 2.51%, and 7.08%, respectively. The reaction rate constants $K_{2}^{\prime }$ decreased by 1.14%, 2.39%, and 6.24%, respectively. The reaction rate constants $K_{3}^{\prime }$ decreased by 3.14%, 11.66%, and 29.15%, respectively. $\alpha_{1}$ and $\alpha_{2}$ increased by 32.68%. As the *n* value decreases gradually, it indicates that increasing the dosage of MgO-based expansive agent inhibits the growth of the product; the decrease in the values of $K_{1}^{\prime }$ and $K_{2}^{\prime }$ suggests that increasing the dosage of MgO-based expansive agent affects the alkalinity of the pore solution, inhibits the growth rate of crystals, affects the time required to reach supersaturation, and reduces the interfacial area. As $\alpha_{1}$ and $\alpha_{2}$ gradually increase, the addition of MgO-based expansive agent on the surface causes the reaction process directly transitions from NG to D at higher hydration levels. This is attributed to the increased hydration level of the entire system due to the addition of MgO-based expansive agent. Under the conditions of this study, as the amount of expansive agent added gradually increases, the hydration level of the reaction system also correspondingly rises.

**Figure 11:**
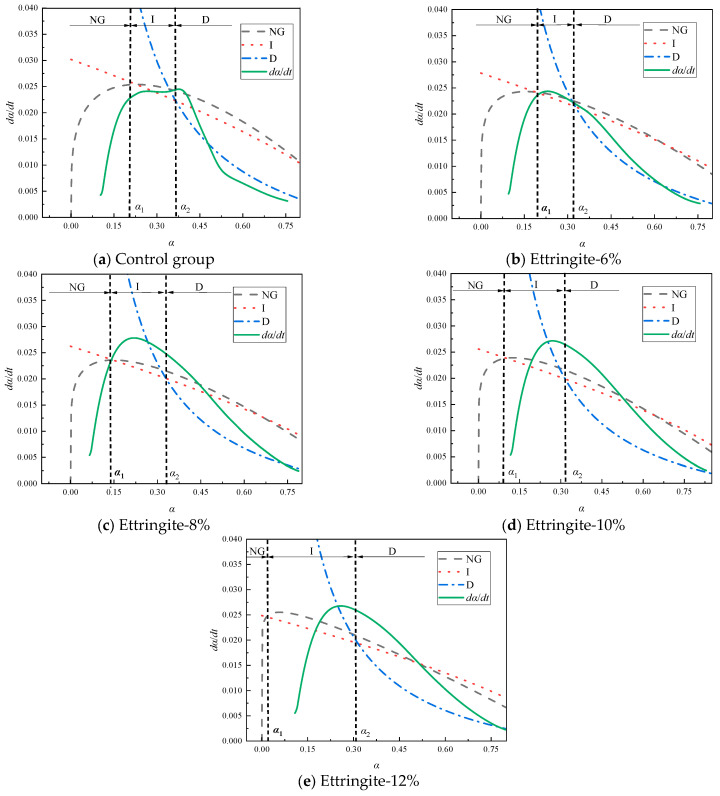
Hydration reaction rate curve of filling materials doped with ettringite-based expansion agent.

Figure 11 shows the simulated curves and corresponding hydration rate curves calculated using the formula under different ettringite-based expansive agent dosage conditions. When using ettringite expansive agents, the hydration reaction undergoes the three stages of NG, I, and D. As the ettringite-based expansive agent is added, the reaction time of the NG stage decreases. Increasing the dosage of the ettringite expansive agent reduces the growth space for hydration products, thereby inhibiting the hydration reaction and causing the NG stage to shrink. The increase in the I stage is attributed to the fact that as the dosage of ettringite expansive agent increases, the reaction rate of the system decreases, and the surface of the particles cannot be fully covered by the products in the short term, reducing the resistance of the hydration reaction and thereby prolonging the transition to the D (diffusion) stage. Overall, the phase boundary reaction stage is significantly influenced by the ettringite-based expansive agent, greatly prolonging the duration of the phase boundary reaction.

Table 6 presents the hydration kinetic parameters of the ettringite-based expansive agent samples. As shown in Table 6, the value of $K_{1}^{\prime }$ is approximately 3.5 times that of $K_{2}^{\prime }$, and approximately 14 times that of $K_{3}^{\prime }$. As the ettringite-based expansive agent content increases, *n*, $K_{1}^{\prime }$, $K_{2}^{\prime }$, $K_{3}^{\prime }$, $\alpha_{1}$, and $\alpha_{2}$ all decrease gradually. Specifically, the reaction order *n* decreased by 1.06%, 3.9%, and 11.64%, respectively. The reaction rate constant $K_{1}^{\prime }$ decreased by 3.47%, 3.87%, and 6.59%, respectively. The reaction rate constant $K_{2}^{\prime }$ decreased by 5.82%, 8.08%, and 10.67%, respectively. The reaction rate constant $K_{3}^{\prime }$ decreased by 4.8%, 11.35%, and 14.41%, respectively. $\alpha_{1}$, and $\alpha_{2}$ decreased by 22.63%, 46.16%, and 90.11%, respectively, and 3.01%, 3.44%, and 5.42%, respectively. Among them, both $\alpha_{1}$ and $\alpha_{2}$ decrease with increasing dosage, indicating that as the dosage of ettringite expansive agent increases, the hydration reaction system requires a higher degree of hydration to transition from the NG process to the I process, while the transition from the I process to the D process requires a lower degree of hydration. This suggests that ettringite-based expansive agents hurt the performance development in the later stages of the hydration process, preventing further improvement in hydration performance. The constants $K_{1}^{\prime }$, $K_{2}^{\prime }$, and $K_{3}^{\prime }$ all decrease with increasing addition amount, primarily because increasing the expansion agent addition amount reduces the peak hydration rate that the filling material can achieve. The trends in $K_{1}^{\prime }$ and $K_{2}^{\prime }$ indicate that the hydration reaction rate and product formation rate of the filling material in this process are relatively fast. The decrease in $K_{3}^{\prime }$ indicates that the increase in ettringite-based expansion agent inhibits the D process.

## 4. Analysis of the Hydration Mechanism in the Middle and Later Stages

### 4.1. XRD Test Results

**Figure 12:**
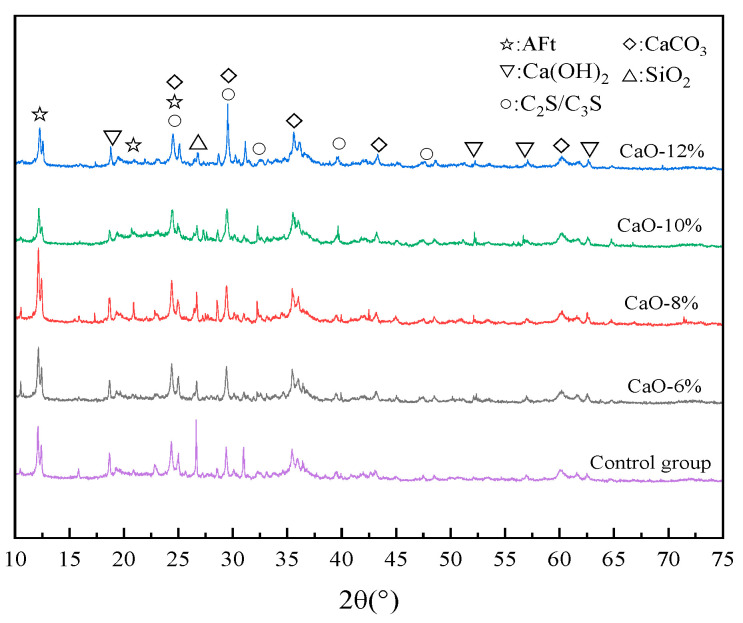
XRD test results of filling materials doped with CaO-based expansion agent.

Figure 12 shows the XRD spectra of samples with CaO-based expansive agent contents of 0%, 6%, 8%, 10%, and 12%. Figure 12 indicates that the diffraction spectra of samples with different contents are similar, suggesting that the types of hydration products formed after the samples have set are stable and essentially the same. The presence of small amounts of C_3_S/C_2_S is due to their hydration primarily occurring in the later stages and not being fully consumed in the short term. The presence of small amounts of SiO_2_ is attributed to slight uneven mixing of the cement clinker, which does not lead to hydration. Both Ca(OH)_2_ and C-S-H exhibit high peak values and large peak areas, indicating they possess high crystallinity and relatively high content.

It can also be seen from Figure 12 that the peak value of CaCO_3_ in the XRD pattern is the highest and increases with an increase in the dosage of the CaO-based expansive agent. This is mainly due to the incomplete reaction of cement and calcium ion diffusion in the filling material. During the secondary reaction process, calcium silicate hydrate and Ca(OH)_2_ are generated. After coming into contact with CO_2_, the reaction as shown in Equation (12) occurs, generating CaCO_3_.

(12) $$\mathrm{Ca}{\left(\mathrm{OH}\right)}_{2}+{\mathrm{CO}}_{2}\to {\mathrm{CaCO}}_{3}+H_{2}O$$

In addition, compared with the control group, samples mixed with the CaO-based expansive agent contained more CaCO_3_, mainly because there were more free Ca^2+^ ions inside the samples, which promoted the formation of CaCO_3_ during the diffusion process.

**Figure 13:**
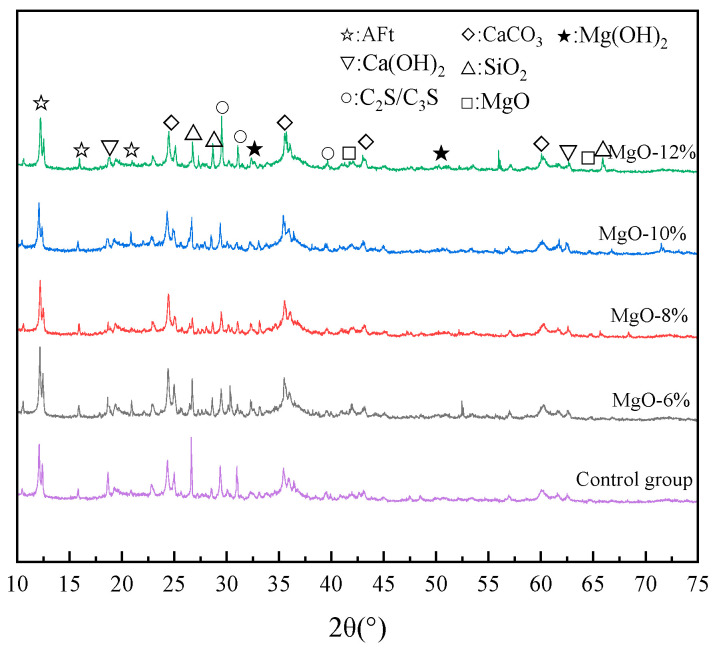
XRD test results of filling materials doped with MgO-based expansion agent.

Figure 13 presents the XRD patterns of the filled samples with MgO expansive agent dosages of 0%, 6%, 8%, 10%, and 12%. Figure 13 shows that distinct diffraction peaks of Ca(OH)_2_, CaCO_3_, C_3_S/C_2_S, AFt, Mg(OH)_2_, and SiO_2_ can be observed in the XRD pattern of the sample. After the introduction of MgO, the characteristic peak of Ca(OH)_2_ showed a decreasing trend, which was attributed to the competitive adsorption mechanism of MgO water molecules, which hindered the hydration of the silicate mineral phase and inhibited the development of Ca(OH)_2_ crystals in the hydration products. This conclusion corroborates the diffraction peak changes of C_3_S/C_2_S. When the MgO content increases to 12%, the unreacted C_3_S/C_2_S significantly increases. Meanwhile, the peak of MgO slightly intensifies as the dosage of the expansive agent gradually increases, but the diffraction peak of Mg(OH)_2_ is exactly the opposite.

**Figure 14:**
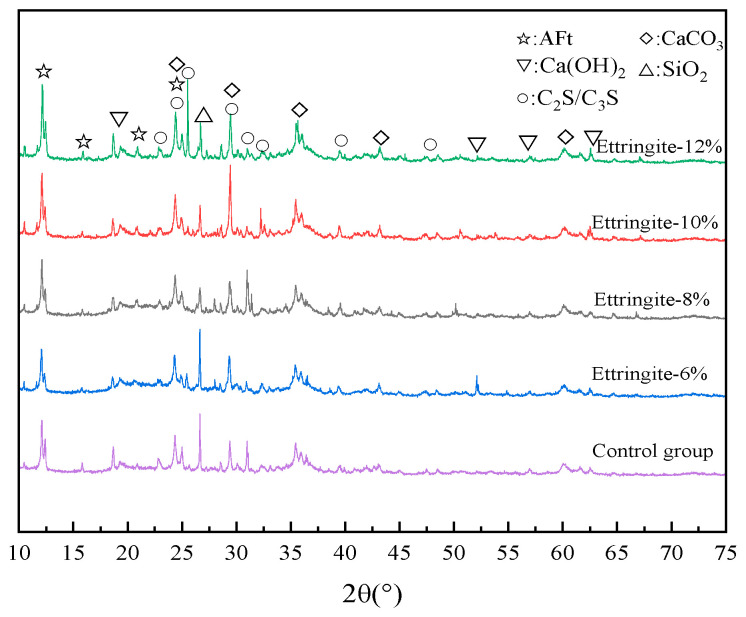
XRD test results of filling materials doped with ettringite-based expansion agent.

Figure 14 shows the XRD spectra of the filled samples with ettringite expansive agent contents of 0%, 6%, 8%, 10%, and 12%. As observed in Figure 14, the diffraction peaks of AFt are distinctly visible in the XRD spectra of the samples, and the intensity of these peaks increases gradually with an increase in admixture content. Analysis suggests that this is due to the secondary hydration of calcium sulfoaluminate, which results in the extensive formation of AFt. AFt is formed during the early hydration stage, and upon complete hydration, it rapidly absorbs water and expands.

### 4.2. TG-DTG Test Results

This section analyzes the late-stage hydration behavior of pre-stressed grouting materials using TG-DTG techniques. As shown by the XRD results from the previous section, the primary hydration products of the three different expansive agents are Ca(OH)_2_ and Mg(OH)_2_. During sample preparation, the filling material samples are ground into powder, resulting in sufficient contact between the sample and air, causing Ca(OH)_2_ and Mg(OH)_2_ to undergo carbonation and form CaCO_3_ and MgCO_3_. Therefore, during the analysis process, the carbonation products must be accounted for. According to literature reviews, the weight loss interval for Mg(OH)_2_ is between 380 and 480 °C, for Ca(OH)_2_ it is between 380 and 480 °C, and for MgCO_3_ it is between 490 and 520 °C. and the weight loss interval for CaCO_3_ is between 565 and 670 °C [40,41,42]. Based on previous research, this study selected the dehydration weight loss interval for Mg(OH)_2_ as 280–380 °C, and then calculated the content of each decomposition characteristic phase under different expansive agents and dosage conditions using Equations (13) and (14), where $M_{Mg{\left(OH\right)}_{2}}$ and $M_{Ca{\left(OH\right)}_{2}}$ respectively represent the contents of Mg(OH)_2_ and Ca(OH)_2_ generated in the hydration reaction; $M_{i{}^{\circ}C}$ represents the percentage of the remaining mass of the sample at *i* °C.

(13) $$M_{Mg{\left(OH\right)}_{2}}=\frac{(M_{380{}^{\circ}C}-M_{280{}^{\circ}C})\times 58}{18}+\frac{(M_{520{}^{\circ}C}-M_{490{}^{\circ}C})\times 84.3}{44.0}$$

(14) $$M_{Ca{\left(OH\right)}_{2}}=\frac{(M_{480{}^{\circ}C}-M_{380{}^{\circ}C})\times 74.1}{18}+\frac{(M_{670{}^{\circ}C}-M_{565{}^{\circ}C})\times 100.1}{44.0}$$

**Figure 15:**
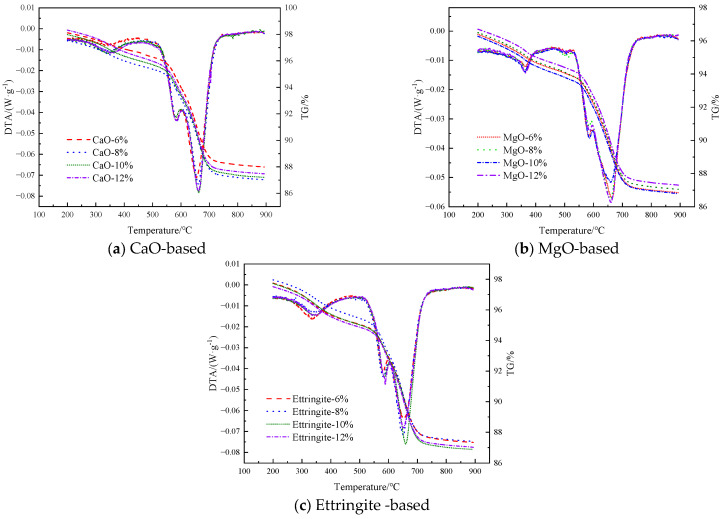
TG-DTA curves of filling materials under different types and dosages of expansion agents.

**Figure 16:**
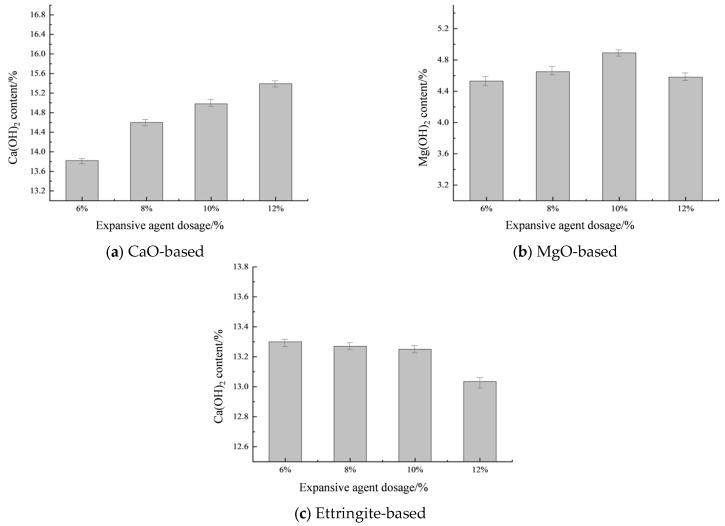
The content of hydration products under different types and dosages of expansion agents.

Figure 15 shows the thermogravimetric-differential thermal analysis (TG-DTA) curves of the filler material under different types of expansive agents and mixing ratios. Based on the TG-DTA curves shown in Figure 15, the contents of Mg(OH)_2_ and Ca(OH)_2_ in the reaction system under different types and proportions of expansive agents were calculated using Equations (13) and (14). The calculation results are shown in Figure 16.

Figure 16 shows the Ca(OH)_2_ and Mg(OH)_2_ content in the filling material under different types and dosages of expansive agents. As shown in Figure 16a, increasing the dosage of CaO-based expansive agent significantly promotes the formation of Ca(OH)_2_ crystals in the hydration system of the filling material, thereby enhancing its expansive capacity. As the CaO expansive agent content increased from 6% to 8%, 10%, and 12%, the Ca(OH)_2_ content increased by 0.78%, 1.16%, and 1.57%, respectively. The Ca(OH)_2_ content does not increase linearly with the increase in CaO expansive agent content, and the extent of its influence decreases with increasing content. Figure 16b shows that when the MgO-based expansive agent content is below 10%, the production of Mg(OH)_2_ is positively correlated with the content, as the free water within the filling material is available for MgO hydration. However, when the content exceeds 10%, the increased temperature within the reaction system promotes the conversion of Ca(OH)_2_, leading to a decrease in OH^−^ ions within the system, making it difficult for Mg(OH)_2_ to nucleate and inhibiting the formation of Mg(OH)_2_ crystals [43]. Figure 16c shows that as the dosage of ettringite-based expansive agent increases, the content of Ca(OH)_2_ crystals in the system decreases gradually. This is primarily due to the presence of Al_2_O_3_ and SO_3_ in the ettringite-based expansive agent, which consumes Ca^2+^ in the system, thereby reducing the content of Ca(OH)_2_ crystals in the system.

## 5. Conclusions

In this study, an isothermal calorimeter was employed to measure the ARCB hydration heat release rate curves of three types of expansive agents: CaO-based, MgO-based, and ettringite-based. Hydration kinetic parameters were calculated based on the Krstulovic–Dabic model. Concurrently, XRD and thermogravimetric analysis methods were integrated to investigate the effects of expansive agent type and dosage on the hydration process of ARCB materials. The conclusions are as follows:

(1) As the dosage of CaO-based expansive agent increases, the cumulative hydration heat rises, and the acceleration phase lengthens. At the same dosage, MgO-based expansive agents exhibit lower heat release than CaO-based agents, a slightly longer induction period, and a slightly higher heat release rate during the decay phase. As the dosage of ettringite-based expansive agents increases, the cumulative hydration heat decreases, the time to reach maximum hydration rate and end the induction phase is slightly earlier, and the heat release rate during the decay phase is lower.

(2) The control group fully undergoes the NG→I→D three-stage process. CaO-based and MgO-based expansive agents cause the filling material to skip Stage I, transitioning directly from NG to D. Ettringite-based expansive agents still undergo three stages, but shorten the NG stage and prolong the I stage.

(3) XRD analysis revealed that increased CaO-based content enhanced the CaCO_3_ diffraction peak, while increased MgO-based content attenuated the Ca(OH)_2_ peak and increased unreacted C_3_S/C_2_S. Increased calcined magnesia-based content strengthened the AFt diffraction peak. TG-DTG analysis indicates that increasing CaO-based content promotes Ca(OH)_2_ formation, Mg (OH)_2_ content showed a positive correlation with MgO-based content ≤ 10%, but was suppressed when content > 10%. Increased calcined magnesia-based content reduced Ca(OH)_2_ content.

In the future, building upon this research, we will delve deeper into the intrinsic mechanisms by which expansive agents regulate the hydration phase transition of ARCB. We will refine quantitative correlation models linking kinetic parameters to the evolution of hydration products.

## Figures and Tables

**Table 1:** The chemical composition of the backfill aggregate.

Aggregate	Chemical Composition (%)							
	SiO_2_	CaO	MgO	Al_2_O_3_	Fe_2_O_3_	SO_3_	K_2_O	Other
Tailings	42.20	3.73	32.71	4.04	12.14	3.37	0.39	1.42

**Table 2:** XRF chemical element analysis of EA.

EA Type	Chemical Element/%							
	SiO_2_	CaO	MgO	Al_2_O_3_	Fe_2_O_3_	SO_3_	K_2_O	Other
CaO-based	2.09	90.89	1.52	0.84	3.81	0.45	0.01	0.39
MgO-based	2.63	2.73	92.25	0.57	1.06	0.28	0.07	0.41
Ettringite-based	12.82	35.18	0.42	5.48	3.57	40.49	0.99	1.05

**Table 3:** Type and dosage of expansion agent.

Name	CaO-Based	MgO-Based	Ettringite-Based
1	6%	6%	6%
2	8%	8%	8%
3	10%	10%	10%
4	12%	12%	12%

**Table 4:** Hydration kinetics parameters of filling materials doped with CaO-based expansion agent.

Sample Number	*n*	$K_{1}^{\prime }$	$K_{2}^{\prime }$	$K_{3}^{\prime }$	Hydration Mechanism	$\alpha_{1}$	$\alpha_{2}$
CaO-6%	1.2052	0.02395	0.00909	0.00168	NG-D	--	0.31262
CaO-8%	1.2167	0.02716	0.00927	0.0017	NG-D	--	0.31062
CaO-10%	1.2259	0.03019	0.01083	0.00207	NG-D	--	0.29659
CaO-12%	1.2382	0.03224	0.01064	0.00215	NG-D	--	0.27499
Control group	1.352	0.0346	0.01006	0.00283	NG-I-D	0.2143	0.3627

**Table 5:** Hydration kinetics parameters of filling materials doped with MgO-based expansion agent.

Sample Number	*n*	$K_{1}^{\prime }$	$K_{2}^{\prime }$	$K_{3}^{\prime }$	Hydration Mechanism	$\alpha_{1}$	$\alpha_{2}$
MgO-6%	1.2513	0.03265	0.00962	0.00223	NG-D	0.30661	0.30661
MgO-8%	1.2128	0.03202	0.00951	0.0023	NG-D	0.31863	0.31863
MgO-10%	1.1641	0.03183	0.00939	0.00249	NG-D	0.34612	0.34612
MgO-12%	1.0994	0.03034	0.00902	0.00288	NG-D	0.40681	0.40681
Control group	1.352	0.0346	0.01006	0.00283	NG-I-D	0.2143	0.3627

**Table 6:** Hydration kinetics parameters of filling materials doped with ettringite-based expansion agent.

Sample Number	*n*	$K_{1}^{\prime }$	$K_{2}^{\prime }$	$K_{3}^{\prime }$	Hydration Mechanism	$\alpha_{1}$	$\alpha_{2}$
Ettringite-6%	1.2044	0.03231	0.00928	0.00229	NG-I-D	0.18236	0.33266
Ettringite-8%	1.1916	0.03119	0.00874	0.00218	NG-I-D	0.14109	0.32264
Ettringite-10%	1.1574	0.03106	0.00853	0.00203	NG-I-D	0.09819	0.32121
Ettringite-12%	1.0642	0.03018	0.00829	0.00196	NG-I-D	0.01803	0.31462
Control group	1.352	0.0346	0.01006	0.00283	NG-I-D	0.2143	0.3627

## Data Availability

The original contributions presented in this study are included in the article. Further inquiries can be directed to the corresponding authors.
